# Cell-free protein synthesis as a novel tool for directed glycoengineering of active erythropoietin

**DOI:** 10.1038/s41598-018-26936-x

**Published:** 2018-06-04

**Authors:** Anne Zemella, Lena Thoring, Christian Hoffmeister, Mária Šamalíková, Patricia Ehren, Doreen A. Wüstenhagen, Stefan Kubick

**Affiliations:** 1 Fraunhofer Institute for Cell Therapy and Immunology (IZI), Branch Bioanalytics and Bioprocesses (IZI-BB), Am Mühlenberg 13, Potsdam, 14476 Germany; 2 University of Potsdam, Karl-Liebknecht-Str. 24-25, Potsdam, 14476 Germany

## Abstract

As one of the most complex post-translational modification, glycosylation is widely involved in cell adhesion, cell proliferation and immune response. Nevertheless glycoproteins with an identical polypeptide backbone mostly differ in their glycosylation patterns. Due to this heterogeneity, the mapping of different glycosylation patterns to their associated function is nearly impossible. In the last years, glycoengineering tools including cell line engineering, chemoenzymatic remodeling and site-specific glycosylation have attracted increasing interest. The therapeutic hormone erythropoietin (EPO) has been investigated in particular by various groups to establish a production process resulting in a defined glycosylation pattern. However commercially available recombinant human EPO shows batch-to-batch variations in its glycoforms. Therefore we present an alternative method for the synthesis of active glycosylated EPO with an engineered O-glycosylation site by combining eukaryotic cell-free protein synthesis and site-directed incorporation of non-canonical amino acids with subsequent chemoselective modifications.

## Introduction

The glycosylation moieties of hEPOs cover up to 40% of the final molecular weight and they have a significant influence on its stability and solubility^1^. Cloned hEPO is available since 1984^2^ and since that time it has been transformed, expressed and purified from mammalian cell lines^3,4^ yeast^5^, insect cells^6^, bacteria^2^ and plants^7^. However, it is the mammalian cell expression that is mostly used to produce recombinant hEPO due to the presence of highly complex glycosylation patterns, which are a crucial factor influencing the pharmacokinetic activity of hEPO *in vivo*^8^. Purified recombinant hEPO (rhEPO) is clinically used to treat anemia caused by specific chronic diseases such as chronic renal failure, chemotherapy, surgery and others^9–11^. The wound-healing effects enlarge the range of therapeutical applications of hEPO to the conditions that are not associated with anemia, such as traumatic brain injury or cardiac diseases^12–14^. The entire process of protein glycosylation is forged within several competing reactions in processing pathways and changes with the cell type, metabolic state, environment and developmental stage. Therefore the final glycan trees differ in monosaccharide composition, structure, type of glycosidic bond and length and might be further modified by sulfation, acetylation, phosphorylation and so forth^15^. In addition a specific glycosylation site might be associated with different glycoforms and not all glycosylation sites must be necessarily occupied. Such a high degree of glycan micro and macro heterogeneity gives rise to a set of glycoforms which expand the proteome diversity far beyond the genetic code and reflects functional diversity required in complex organisms. Endogenous and recombinant forms of hEPO are produced as complex mixtures of glycoforms^15^. Under *in vivo* conditions, individual hEPO glycoforms show different levels of biological activity and biostability.

As a promising alternative to *in vivo* produced and chemically synthesized hEPO, we present the potential of cell-free glycoprotein synthesis. In this report a eukaryotic translationally active lysate derived from cultured cells of *Spodoptera frugiperda* (*Sf*21) with endogenous ER-derived membraneous structures termed microsomes, was utilized for the experiments^16,17^. Generally, the mechanism of glycosylation in mammalian cells and invertebrates proceeds through similar pathway, being initiated by *en bloc* transfer of a conserved 14-mer oligosaccharide (Glc_3_Man_9_GlcNAc_2_) onto NXS/T/C motifs within newly synthesized polypeptides, followed by a subsequent step-wise sugar trimming and sugar adding. However, while mammalian N-glycans are typically multiply branched, complex and terminally sialylated, insect cells generate large numbers of much simpler, high-mannose type and paucimannose N-glycans. The chosen *Sf*21 lysate is, in contrast to prokaryotic lysates, endotoxin-free and therefore a suitable system for the production of pharmaceutically relevant proteins. The synthesis of the protein of interest starts immediately after addition of a template, amino acids, energy and an energy regeneration system.

## Materials and Methods

### Lysate preparation

Translationally active insect cell lysates were produced as described previously^18^.

### Generation of template DNA

The hEPO encoding gene sequence was cloned into the pIX2.0 vector (RiNA GmBH, Berlin, Germany), and it’s native signal sequence was replaced by the melittin signal sequence of the honey bee (MKFLVNVALVFMVVYISYIYAD) which has been shown previously^19^) to induce an efficient translocation into the microsomes, leading to formation of recombinant hEPO (EPO).

Generation of a mutant template DNA containing an internal *amber*-stop codon (EPO-Amb) was achieved via vector-PCR (V-PCR). PCR-primers differing from the original sequence by replacement of Ser 153 (native O-glycosylation site) with an *amber*-stop codon were designed (forward primer: 5′ CCTCCAGATGCGGCC**TAG**GCTGCTCCACTCCGA 3′, reverse primer: 5′ TCGGAGTGGAGCAGC**CTA**GGCCGCATCTGGAGG 3′) and combined with 1 ng/µL of pIX2.0-EPO plasmid DNA, 10 µL HiFi-buffer (Qiagen, Hilden, Germany), and 0.05 U/µL HiFi-Polymerase (Qiagen, Hilden, Germany) in a 50 µL PCR-reaction (denaturation for 60 sec at 94 °C, annealing for 60 sec at 63 °C, elongation for 10 min at 68 °C). After 19 cycles, the resulting PCR-product was treated with the restriction endonuclease DpnI (10 U/µL, 60 min, 37 °C, NEB, Ipswich MA, U.S.A.) digestion (60 min, 37 °C). DNA was subsequently purified using a Dye-Ex 2.0 Spin Kit (Qiagen, Hilden, Germany). Successful *amber*-stop codon introduction lead to an additional StyI restriction site, resulting in three (3276, 318 and 217 bp) instead of two (3276 and 535 bp) cleavage products and was verified by agarose gel electrophoresis. Constructs were transformed and propagated using electrocompetent *E. coli* cells (*E. coli* JM109, NEB).

Template DNA for the orthogonal tRNA run-off transcription was generated using a PCR reaction (0.01 ng/µL plasmid DNA, dNTPs 0.2 mM each, MgCl_2_ 2.5 mM, Taq-polymerase 0.025 U/µL, 1x Taq-buffer, denaturation for 30 sec at 95 °C, annealing for 30 sec at 52 °C, elongation for 30 sec at 72 °C, 30 cycles, and final elongation for 10 min at 72 °C) together with a specific O-methyl primer pair (0.5 µM each). After PCR, the product was purified using Qiaquick PCR-Purification Kit (Qiagen, Hilden, Germany), analyzed via agarose gelelectrophoresis and used for run-off transcription (see below).

### Generation of orthogonal components

The orthogonal aminoacyl-tRNA synthetase (eAzPheRS) specific for p-propargyloxyphenylalanine (pPa) and p-azido-L-phenylalanine (AzF) was synthesized in the “RTS500 ProteoMaster *E. coli* HY Kit” (Biotechrabbit, Henningsdorf, Germany). Protein synthesis was induced by IPTG and *de novo* synthesized aminoacyl-tRNA Synthetase was purified via Strep-tagII by affinity chromatography on *Strep* Tactin superflow columns (IBA, Goettingen, Germany). Fractions containing the purified enzyme were pooled and loaded onto a NAP25 column (GE Healthcare, Freiburg, Germany), equilibrated with synthetase storage buffer (50 mM HEPES, 10 mM KOAc, 1 mM MgCl_2_, 4 mM DTT, 0.02% NaN_3_, pH 7.6). Eluates were concentrated in an Amicon Ultra-4 Centrifugal Filter Device (cut-off 10 kDa, Millipore, Billerica MA, U.S.A.) at 4,000 × g and 25 °C to a final concentration of 15–20 mg/mL. Aliquots of the concentrated aminoacyl-tRNA synthetase were stored at −80 °C.

Specific 5′ run-off transcripts of suppressor tRNAs were transcribed *in vitro* over night in a batch format at 37 °C, using a PCR product as a DNA template (final concentration: 8 µg/mL). The T7 polymerase (1 U/µL) based reaction was performed using the EasyXpress Insect Kit II (Qiagen, Hilden, Germany) according to the manufacturer’s instructions, but using an NTP-mixture without cap analogues. tRNAs were purified via phenol-chloroform extraction deploying TRIzol-reagent (Life Technologies, Carlsbad CA, U.S.A.) according to the manufacturer’s protocol. Precipitated tRNA was subsequently resuspended in ultra pure water and stored at −80 °C.

### Cell-free synthesis of EPO

Target protein synthesis was achieved using pIX2.0 plasmids in a linked system based on translationally active *Sf*21-lysates.

Transcription reactions were carried out similarly to the previously described tRNA transcription, using 60 µg/mL of purified plasmid DNA, NTPs and cap analogues. Generated mRNA was purified by gel filtration using either DyeEx2.0 spin columns (Qiagen, Hilden, Germany) or Nap5 columns (GE Healthcare, Freiburg, Germany) according to the manufacturer’s instructions.

The standard batch translation reaction mixture, ranging in volumes from 5–500 µL, contained 40% (v/v) *Sf*21 lysate, approximately 400 µg/mL coding mRNA, canonical amino acids (200 µM), ATP (1.75 mM) and GTP (0.45 mM). For monitoring protein quality and quantity reaction mixtures were supplemented either with ^14^C-labeled leucine (310.0 mCi/mmol, PerkinElmer, Waltham MA, U.S.A.) or ^14^C-labeled mannose (55 mCi/mL, ARC Inc., St. Louis MO, U.S.A.). Translation reactions were operated at 27 °C and 500 rpm in a thermomixer (Thermomixer comfort, Eppendorf, Hamburg, Germany) for 90–120 minutes. As negative controls, standard translation reactions were performed without the addition of mRNA (no-template control, NTC).

#### Repetitive synthesis for MS analysis

EPO was prepared in a batch mode in a final volume of 100 µL. After each translation cycle, the original set of microsomes was collected by centrifugation (20 min, 15,000 × g). The microsomal pellet resulting from centrifugation was resuspended in a new aliquot of translation mixture containing fresh components required for translation, such as a fresh mRNA aliquot and fresh lysate deprived of microsomes by a centrifugation step (10 min, 4 °C, 16,000 × g). Each cycle corresponded to the incubation period of 90–120 min at 27 °C and 500 rpm in a thermomixer (Thermomixer comfort, Eppendorf, Hamburg, Germany). After the third cycle, vesicles were collected by centrifugation (20 min, 15,000 × g) and the pellet was subjected to a standard acetone precipitation protocol for 1 h to remove salts. Precipitated proteins were recovered by centrifugation (20 min, 15,000 × g) and were briefly dried in a vacuum centrifuge (Eppendorf, Hamburg, Germany).

#### Orthogonal translation

For the site-directed incorporation of p-azido-L-phenylalanine (AzF) as well as p-propargyloxyphenylalanine (pPa), the corresponding ncAA (2 mM) and the orthogonal tRNA/tRNA synthetase pair tRNATyr_CUA_ (2 µM)/eAzPheRS (2 µM) were added to a standard batch translation reaction mixture, using *Sf*21-lysate at a final concentration of 35%. The final volume of the translation reaction mixture was 20–100 µL. Repetitive translation was performed in analogy to the previously described synthesis of EPO for MS analysis.

#### Vesicle release

For chemoselective labeling as well as for cell-based activity assays, newly synthesized EPO had to be released from the lumen of the ER-based microsomes (Supplementary S1). The microsomal fraction was collected by centrifugation (10 min, 4 °C, 16,000 × g), and proteins were released from the vesicles at 4 °C by pellet resuspension in the initial volume of the reaction with PBS + 0.1% n-Dodecyl β-D-Maltopyranoside (DDM, Qiagen, Hilden, Germany). The release was enhanced by vigorous shaking (Vibrax VXR, IKA, Staufen, Germany). Remaining insoluble structures were removed by a second centrifugation step (10 min, 4 °C, 16,000 × g). The supernatant (SNVF) containing translocated and released EPO was directly used in the EPO-activity assay. Alternatively released EPO-Amb was subjected to chemoselective labeling beforehand.

### Chemoselective Labeling

The chemoselective copper dependent azide-alkyne cyclo addition of pPa was performed in PBS with 250 µM 3[tris(3-hydroxypropyltriazolylmethyl)amine (ThPTA, Sigma-Aldrich, St. Louis MO, U.S.A.), 5 mM sodium ascorbate (Sigma-Aldrich, St. Louis MO, U.S.A.), 50 µM copper sulfate. As reacting agents for pPa, either 10 µM Cy 5-azide (Cy5, Lumiprobe, Hannover, Germany) or 10 mM 10 kDa PEG-azide (PEG10k, Iris Biotech, Marktredwitz, Germany) were used, respectively. The reaction was performed in the dark for 2 h at 25 °C.

Chemoselective Staudinger ligation of AzF was carried out by addition of 10 µM DyLight 650-Phosphine (ThermoFisher Scientific, Waltham MA, U.S.A.) and incubation in the dark for 2 h at 25 °C.

### EPO activity assay

UT-7 (Leibnitz-Institut DSMZ, Braunschweig, Germany, DSMZ-No. ACC-137) cells expressing the hEPO receptor were cultivated in 80% MEM (Gibco, Life Technologies, Carlsbad CA, U.S.A.) +20% FCS (Biochrom GmbH, Berlin, Germany), containing 30 µg/mL Gentamycin (Carl Roth GmbH +Co. KG, Karlsruhe, Germany) and 5 ng/mL GM-CSF (Sigma-Aldrich, St. Louis MO, U.S.A.). Incubation was carried out in a 24-well plate (TPP, Trasadingen, Switzerland) in a CO_2_ incubator (Binder, Tuttlingen, Germany) at 37 °C and 5% CO_2_. Cell density was kept between 0.3 and 1.0 × 10^6^ cells/mL. For the activity assay, growing cells were transferred into fresh medium without GM-CSF in final densities of 0.3 × 10^6^ cells/mL. 5–10 µL of fractionated and released cell-free synthesized EPO was applied as repeat determinations on the new cell cultures. Deployed volumes of labeled EPO-analogues were adapted regarding the increased sample volumes. NTC-reactions were applied as negative controls and NTC-reactions supplemented with GM-CSF or recombinant hEPO (Sigma-Aldrich, St. Louis MO, U.S.A., #H5166-10 UG) were used as positive controls. Cell growth was monitored over several days by Trypan blue staining in Thoma-new cell counting chambers (Hirschmann, Eberstadt, Germany) and light microscopy imaging. Total living cell concentrations were estimated by averaging 6 counted aliquots of each sample, followed by median calculation of the 2 replicas.

### Deglycosylation assay

Protein N-glycosylation was probed using PNGase F (peptide N-glycosidase F, NEB, Ipswich MA, U.S.A.) or Endo H (endoglycosidase H, NEB, Ipswich MA, U.S.A.) on ^14^C-labeled EPO. The assay was performed according to the manufacturer’s protocol and the results were analyzed by 1D-SDS-PAGE.

### Protein quantification using hot TCA precipitation

The yield of cell-free synthesized EPO was determined by hot trichloroacetic acid (TCA) precipitation and liquid scintillation quantification as described previously^18^.

### 1D-SDS-PAGE and autoradiography

1D-SDS-PAGE was performed with NuPAGE® and Bolt SDS-PAGE gel electrophoresis systems (ThermoFisher Scientific, Waltham MA, U.S.A.). Aliquots of 5 µL standard translation mixtures were subjected to cold acetone precipitation for 1 hour. Precipitates were collected by centrifugation at 16,000 × g for 10 min at 4 °C. The pellet resulted from centrifugation was resuspended in 20 µL of 1 × LDS sample buffer and loaded on 10% Bis-Tris mini-gels (ThermoFisher Scientific, Waltham MA, U.S.A.). The electrophoretic run was set for 35 min at constant 200 V. Immediately prior to fluorescence imaging, gels were incubated in MQ-H_2_O-Methanol (1:1) for 10 minutes.

Bis-Tris gels with separated ^14^C labeled proteins were stained with Simply Blue^TM^ SafeStain (ThermoFisher Scientific, Waltham MA, U.S.A.), dried for 60 min at 70 °C (Unigeldryer 3545D, UniEquip, Martinsried, Germany) and exposed to phosphor screens for at least 48 hours. Bands of labeled proteins were visualized with phosphor imaging technique (Amersham Typhoon RGB, Typhoon Trio+, Image Eraser, storage phosphor screens and cassettes, ImageQuant TL software, all from GE Healthcare, Little Chalfont, Buckinghamshire, U.K.).

### Fluorescence imaging

Cy5 and DyLight phosphine-650 labeled EPO-Amb were visualized using a fluorescence scan technique on a Typhoon Trio imager (extinction: 633 nm, emission 670 nm, photomultiplier 600) directly after SDS-gel separation.

### 2D-SDS-PAGE

1^*st*^ *dimension*, Isoelectric focusing (IEF): After the third cycle of EPO repetitive synthesis, the translation mixture was fractionated into soluble and microsomal fraction by centrifugation (15,000 × g, 20 min). The vesicular fraction containing EPO was subjected to cold acetone precipitation for a time period of 1 hour. The pellet was resuspended in 40 µL of 2D-SDS-PAGE rehydration buffer (7 M Urea, 2 M thio-urea, 4% CHAPS, 0.5% ampholytes pI range 6–11). Destreak reagent (GE Healthcare, Little Chalfont, Buckinghamshire, UK) was added to the rehydration buffer as a reducing agent. Samples were incubated in rehydration buffer overnight to facilitate the protein resolubilization. An IPG strip 6–11 (ThermoFisher Scientific, Waltham MA, U.S.A.) of 11 cm length was rehydrated overnight using the same rehydration buffer as the one used for sample solubilization. Samples were loaded on the anodic part of the strip using a cup-loading technique. IEF was performed in Ettan IPGphor3 (GE Healthcare, Little Chalfont, Buckinghamshire, UK) at 20 °C with total 12–15,000 kVh applied to the strip. Paper wicks were changed several times during the run. After the IEF completion, the reduction and alkylation of proteins was performed on the strip by incubation of the strip in equilibration buffer (8 M urea, 4% SDS, 150 mM tris pH 8, 20% glycerol) containing 2% dithiotreitol (DTT) (Sigma-Aldrich, St. Louis MO, U.S.A.) for 15 min followed by a 15 min incubation in equilibration buffer containing 3% iodacetamide (IAA, Sigma-Aldrich, St. Louis MO, U.S.A.).

2^*nd*^ *dimension*, SDS-PAGE was performed using a vertical midi SDS-PAGE system (Criterion, Bio-Rad laboratories, Hercules CA, U.S.A.). The strip was loaded on 4–12.5% tris-glycine gradient gel (Bio-rad laboratories, Hercules CA, U.S.A.) and ran for 1 hour at constant voltage of 200 V. Gels were stained using Simply Blue coomassie staining solution (ThermoFisher Scientific, Waltham MA, U.S.A.).

### Proteolytic digestion and nano-LC/MS/MS of glycosylated EPO

The band corresponding to the translated protein was excised from the gel and subjected to in-gel trypsin digestion following the standard protocol. Briefly, the gel pieces containing the protein of interest were washed with water and destained by incubation cycles in a mixture of 50% acetonitrile and 25 mM ammonium bicarbonate (ABC) buffer. Destained gel pieces were incubated with 100% acetonitrile followed by subsequently completely drying in a vacuum centrifuge. Dried pieces were transferred on ice and incubated with the trypsin solution (12.5 ng/µL, Promega, Fitchburg FI, U.S.A.) in 50 mM ABC (pH 8) for half an hour. The excess of trypsin solution was discarded, the gel pieces were covered with 50 mM ABC and were subjected to overnight incubation at 37 °C. Tryptic peptides were extracted from the gel pieces as previously described by Kolarich *et al*.^20^ to enhance glycopeptide extraction. The pool of extracted peptides was dried in a vacuum centrifuge for 1 minute. The resulting pellet was resuspended in 25 µL of 100 mM TrisHCl buffer (pH 8)/10 mM CaCl_2_. Chymotrypsin (0.5 µg, Promega, Fitchburg FI, U.S.A.) was added to the solution and the sample was incubated for 4 hours at 25 °C and 500 rpm. The reaction was terminated by addition of formic acid in a final concentration of 0.1%. Afterwards the peptides were separated by liquid chromatography and analyzed by nano-LC-MS/MS technique.

Nano-LC/MS/MS analysis of peptides resulting from the double digestion was performed on nano-Ultimate 3000 RSLC (Dionex, Amsterdam, the Netherlands) interfaced with an Amazon speed ETD mass spectrometer (Bruker, Bremen, Germany) equipped with captive spray ionization source. Peptide samples of 20 µL were injected using the nano-flow pump. Peptides were first trapped on a trapping column (PepMap™ C18 column, 75 µm I.D. × 20 mm, 3 µm, 100 Å, Dionex, Amsterdam, the Netherlands). After back-flushing from the trapping column, the sample was loaded on a 75 µm I.D. × 250 mm C18 reverse-phase column (Dionex, Amsterdam, the Netherlands). Separation was performed using a linear gradient of 0.5% solvent B (0.1% formic acid in water/80% acetonitrile) increased per minute at a constant flow rate of 300 nL/min. The nano-LC column was kept at 40 °C. To generate a stable electrospray, the capillary voltage was set on 1,300 V. Using data dependent acquisition, 10 multiply charged ions with intensities above the absolute threshold of 25,000 were automatically selected for fragmentation. The target mass was set on 1,200 m/z. MS analysis was performed in the positive ion mode within mass range 400–1,500 m/z using enhanced resolution (8,100 m/z s^−1^) and ultrascan (32,500 m/z s^−1^). Each spectrum corresponded to the average of three scans. External calibration was performed before the nano-LC runs using the Bruker calibration mixture. MS/MS analysis was performed using collision-induced dissociation fragmentation (CID) alternating with electron transfer dissociation fragmentation (ETD) mode in a dynamic mass range starting from m/z 100 up to a mass range corresponding to the 3x m/z of the precursor. The fragmentation amplitude in CID fragmentation was set to 70% to fragment eluting peptides. For ETD fragmentation, the amount of fluoranthene radical anions transferred to the ion trap was monitored and set to the value recommended by the manufacturer of 500,000 by adjusting the reactant accumulation time to a value of 10 ms while reaction time corresponded to 100 ms.

## Results

**Figure 1:**
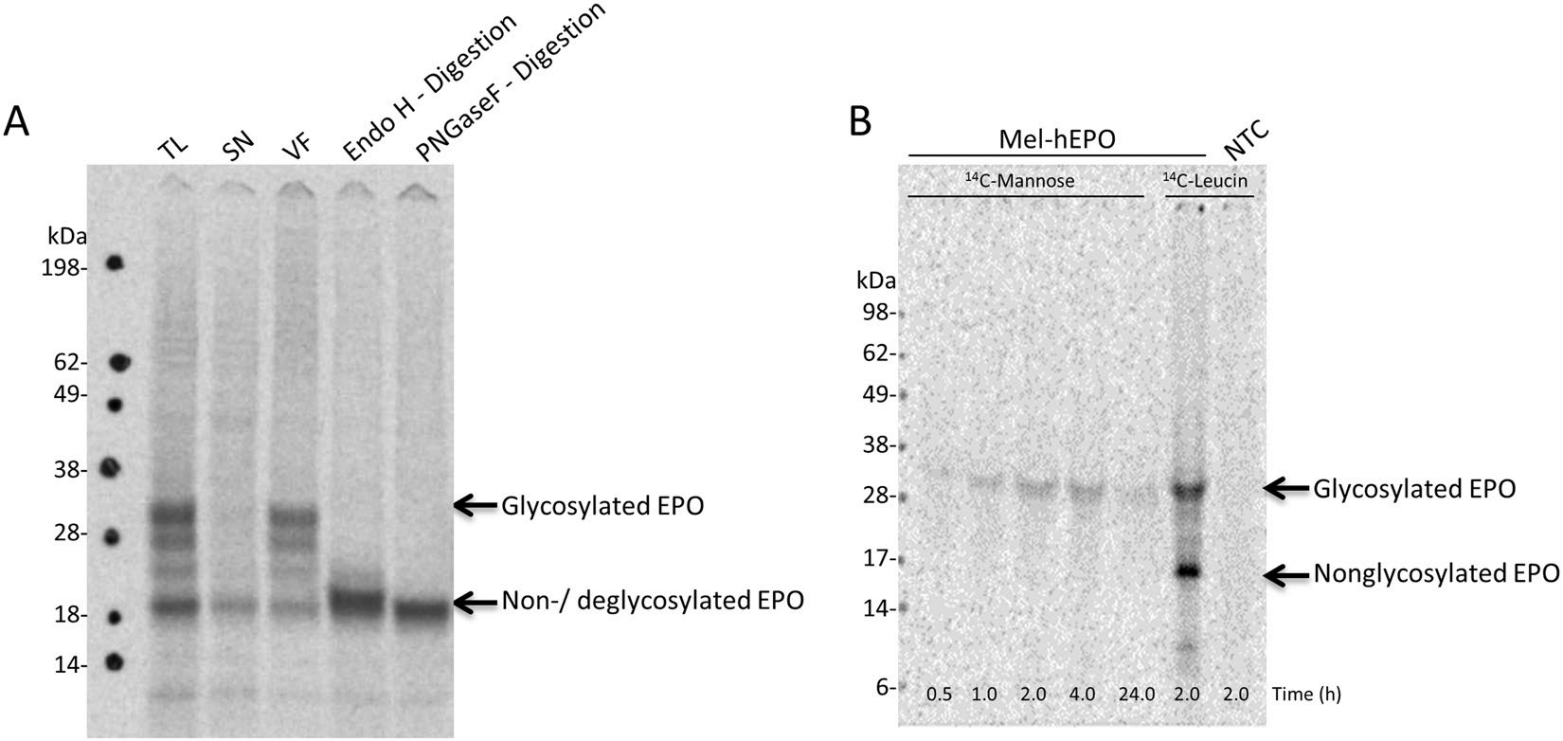
Glycosylation of cell-free synthesized erythropoietin (EPO) by 1D-SDS-PAGE followed by autoradiography. (**A**) Monitoring of translocation and glycosylation of cell-free synthesized ^14^C-labeled EPO. Lane 1: EPO in translational mixture (TL), lane 2: EPO in soluble fraction (SN), lane 3: EPO in vesicular fraction (VF), lane 4: EPO after treatment with EndoH, lane 5: EPO after treatment with PNGaseF. The upper EPO bands correspond to the glycosylated protein, whereas the lower band corresponds to the unglycosylated (lane 1–3) or deglycosylated (lane 4 and 5) protein. Different molecular weight between Endo H and PNGase F digestion results from the different cutting of the enzymes. (**B**) Time-dependent cell-free synthesis of EPO supplemented with free ^14^C-mannose (lane 1: 0.5 hour; lane 2: 1 hour; lane 3: 2 hours; lane 4: 4 hours; lane 5: 24 hours). ^14^C-leucine-supplemented translation reactions of EPO (lane 6: 2 hours) and without template (lane 7: 2 hours) were used as control reactions.

The autoradiography of cell-free synthesized EPO (Fig. 1) shows distinct bands suggesting coexistence of two populations of EPO in the reaction mixture. The lower band with an apparent MW corresponding to approximately 20 kDa is assigned to full length EPO which carries no glycosylation (MW_calc_ = 21 kDa). The upper bands exhibited an apparent MW of 30 kDa and can be assigned to glycosylated EPO. Fractionation of the translational mixture (TL) into the soluble (SN) and vesicular fraction (VF) (Fig. 1) demonstrates that only EPO originating from the microsomal fraction has an increased MW presumably due to glycosylation. This is in agreement with our assumption that the microsomes are ER-derived. The identity of EPO being N-glycosylated is confirmed by a deglycosylation assay with Endo H and PNGaseF (Fig. 1A). The endopeptidase PNGase F cleaves specifically the bond between the asparagine residue of the peptide backbone and the innermost glucose-N-acetylamine of the N-glycan resulting in a loss of the upper band of EPO in the autoradiogram whereas Endo H cleaves the bond between the mentioned N-acetylglucosamines resulting in a distinct band migrating at slightly higher apparent molecular weight in comparison to the PNGaseF digestion.

Labeling of EPO sugar moieties with ^14^C-mannose, one of the building blocks of the glycan-precursor, is presented in Fig. 1B. In contrast to ^14^C-leucine labeled EPO, we observe only one distinct band at an apparent MW of approximately 30 kDa, corresponding to the population of glycosylated protein. Detection of the ^14^C-mannose attached to EPO shows that the glycans bound to the newly synthesized proteins do not originate exclusively from rigid sugar blocks already pre-synthesized in the time before lysate preparation. In fact these precursors are still continuously formed within the lysate, by incorporation of free mannose under cell-free conditions.

**Figure 2:**
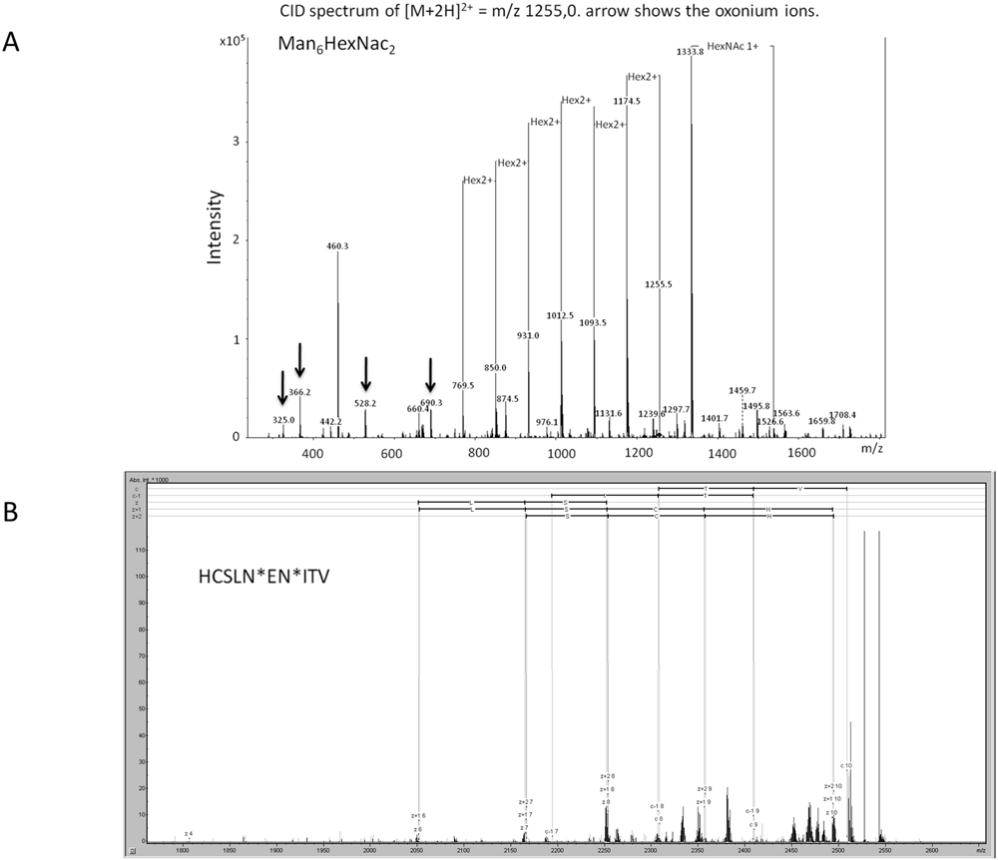
Glycan-identification of cell-free synthesized erythropoietin (EPO) by mass spectrometry (MS). Collision-induced dissociation fragmentation CID; (**A**) and electron transfer dissociation fragmentation ETD; (**B**) Tandem MS spectrum of doubly charged HCSLNEN_Man6HexNac2_ITV glycopeptide of *m/z* 1255 derived from EPO in gel digestion. CID fragmentation resulted in extensive glycan cleavage allowing for glycan structure assignment. ETD fragmentation resulted in peptide backbone cleavage and allows for amino acid sequence assignment.

Glycan structures attached to cell-free synthesized EPO were analyzed in detail using nano-LC/MS/MS after separation of *de novo* synthesized EPO from the remaining microsomal proteins. Separation was performed using the 2D-SDS-PAGE technique with the first dimension carried out in a basic pI gradient of pH 6–11 (pI of EPO = 8.6). The precise position of the EPO band on the 2D-SDS-PAGE was determined by merging the autoradiography picture of ^14^C-leucine radiolabeled EPO with the coomassie stained gel (Supplementary S2). Peptides were separated by liquid chromatography after proteolytic digestion and analyzed by mass spectrometry using two complementary dissociation techniques, namely CID and ETD. CID, a low energy impact technique, generates characteristic oxonium ions (originating from the cleavage of the glycosidic bonds of glycans) and fragment ions originating from the neutral loss of sugar residues which build up the glycan. Only minimal fragmentation of the peptide backbone is usually observed in CID. Thus, from CID spectra, detailed glycan structures can be derived. As an example, CID MS/MS spectrum of doubly charged EPO glycopeptide (m/z 1255) derived from the protein digestion is shown in Fig. 2A. An extensive fragmentation of the glycan antennary structure is reflected by the presence of characteristic glycopeptide diagnostic signals at m/z 325 (Hex 2), m/z 366.1 (Hex 1 HexNAc 1), m/z 528.2 (Hex 2 HexNAc 1) and m/z 690.3 (Hex 3 HexNAc 1). From the successive neutral losses of 162 Da corresponding to the sugar building blocks, the glycan composition can be reconstructed. It can be concluded from the spectrum, that the ion precursor underwent the loss of 6 hexoses before the first HexNac was cleaved off. In this CID spectrum, the second HexNac was not cleaved off and is still attached to the peptide. The glycan structure attached to this peptide contains 6 hexoses and 2 HexNac (Man_6_HexNac_2_). The experimental MW of the “naked” peptide after subtracting the MW of glycan corresponds to 1129.70 Da. This value can be fitted to the theoretical monoisotopic MW of EPO_31–40_ (sequence HCSLNENITV) with the consensus glycosylation site at Asn38 (MW = 1129.52 Da). The increase of the experimental value of 1 Da in reference to the theoretical MW could be explained as a result of asparagine deamidation, a modification which is frequently occurring in proteins and peptides. Fragmentation of the same glycopeptide using the ETD technique is shown in Fig. 2B. In contrast to CID, ETD fragmentation targets the peptide backbone, yielding preferentially *c* and *z* fragments, from which the peptide amino acid sequence and the site of glycosylation can be derived.

ETD fragmentation suffers from known drawbacks, namely low fragmentation efficiency and major reaction pathway being the charge reduction instead of preferred fragmentation-reaction pathway. Despite of this, the generated *c* and *z* ions were of quality good enough to fit the experimentally obtained amino acid sequence with the sequence predicted on the base of the MW of the naked peptide obtained from CID fragmentation of glycopeptides.

Using such an approach of combining the information obtained from CID and ETD techniques, the glycan structures attached to the remaining two consensus glycosylation sites of EPO (Asn24 and Asn83) were also elucidated (Table 1). The glycan pattern in cell-free synthesized EPO using the *Sf*21 lysate corresponds to the high mannose-type structures HexNac_2_(Man)_5–8_, which is in good agreement with the data regarding the insect glycosylation derived from *Drosophila melanogaster (generus Diptera)*^21^. Additionally, the identity of glycosylated residues in cell-free synthesized EPO is in agreement with the Uniprot database knowledge. No O-glycosylation was found in our experiments.

**Table 1:** Glycan composition of the different N-glycosylations within cell-free synthesized EPO, identified by nano-liquid chromatography mass spectrometry based on combined information from collision-induced dissociation and electron transfer dissociation fragmentation.

residue	Peptide sequence	MW_exp_ (Da)	Mw_theor_ (Da)	Glycan composition
N38	HCSLN_deam_EN*ITV	1129.7	1129.52	Man_7_HexNAc_2_ Man_6_HexNAc_2_ Man_5_HexNAc_2_
N24	EAEN*ITTGC_carb_AE	1193.5	1193.49	Man_8_HexNAc_2_ Man_7_HexNAc_2_
N83	LVN*SSQPWEPLQ_deam_L	1510.7	1510.77	Man_8_HexNAc_2_ Man_7_HexNAc_2_ Man_6_HexNAc_2_ Man_5_HexNAc_2_

**Figure 3:**
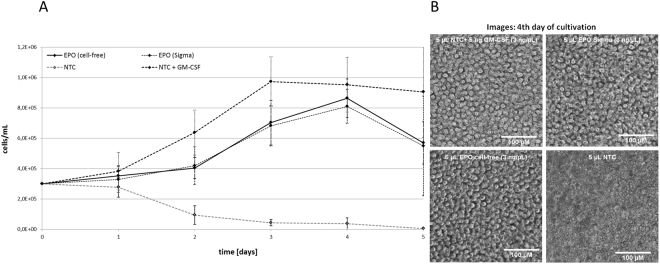
Cell-based activity assay of erythropoietin (EPO). (**A**) Growth curves of UT-7 (DSMZ-No. ACC-137) cell-line supplemented with cell-free synthesized EPO (continuous line), cell-free no-template-control (NTC, grey-dashed line), NTC + GM-CSF (dash-dot line) and commercially available hEPO (Sigma-Aldrich, #H5166-10 UG, dotted line). The concentration of cell-free produced EPO was adjusted according to the total protein concentration determined by TCA-precipitation. EPO as well as commercially purchased hEPO induced cell-growth within 4 days of cultivation. The NTC lead to a continuous decrease of living cells. (**B**) Analysis of UT-7 cells by light microscopy. Cells were visualized after four days of cultivation.

The proliferation activity of different growth factor samples was studied by incubation with the UT-7 human cell line established from bone marrow diagnosed with acute myeloid leukemia^22^. Cell-growth of this specific cell-line can be propagated by GM-CSF, interleukin 3 and erythropoietin. Samples of cell-free synthesized, glycosylated EPO were released from the microsomal fraction with PBS + DDM (0.1%) and total volumes of 5 µL were directly applied on growing cell-cultures. As a negative control, equal amounts of identically treated NTCs were added to the cells. Equivalent samples were supplemented with either commercially available hEPO (f.c. 3 ng/µL) or GM-CSF (f.c. 1 ng/µL) and used as positive controls. Monitoring cell-growth for 5 days resulted in growth curves presented in Fig. 3A. As expected, the application of NTC harboring additional GM-CSF resulted in an intense cell proliferation over the first 3 days with a maximum density of approximately 1 × 10^6^ living cells/mL. This initial growth followed by a constant decrease in living cells after the third day correlates well with previously published results^22^. Application of the negative control resulted in a continuous dieback of the cell culture. Cultures supplemented either with cell-free produced EPO or commercially available hEPO displaced significant cell proliferation as well. As expected, supplementation with cell-free produced EPO as well as commercially available hEPO showed minor effects on culture growth compared to the addition of GM-CSF^22^. Constant cell growth was monitored up to 4 days of cultivation with a maximum of approximately 8.5 × 10^5^ living cells/mL. After day 4, the total cell number declined about 25% within 24 h in both samples. Light microscopic images from the 4^th^ day of cultivation (Fig. 3B, right) confirm the cell proliferation in all samples where a growth factor (EPO, hEPO or GM-CSF) was supplemented, indicating a successful cell-free synthesis of active EPO. The NTC without any growth factor revealed essentially dead cells and cell debris.

**Figure 4:**
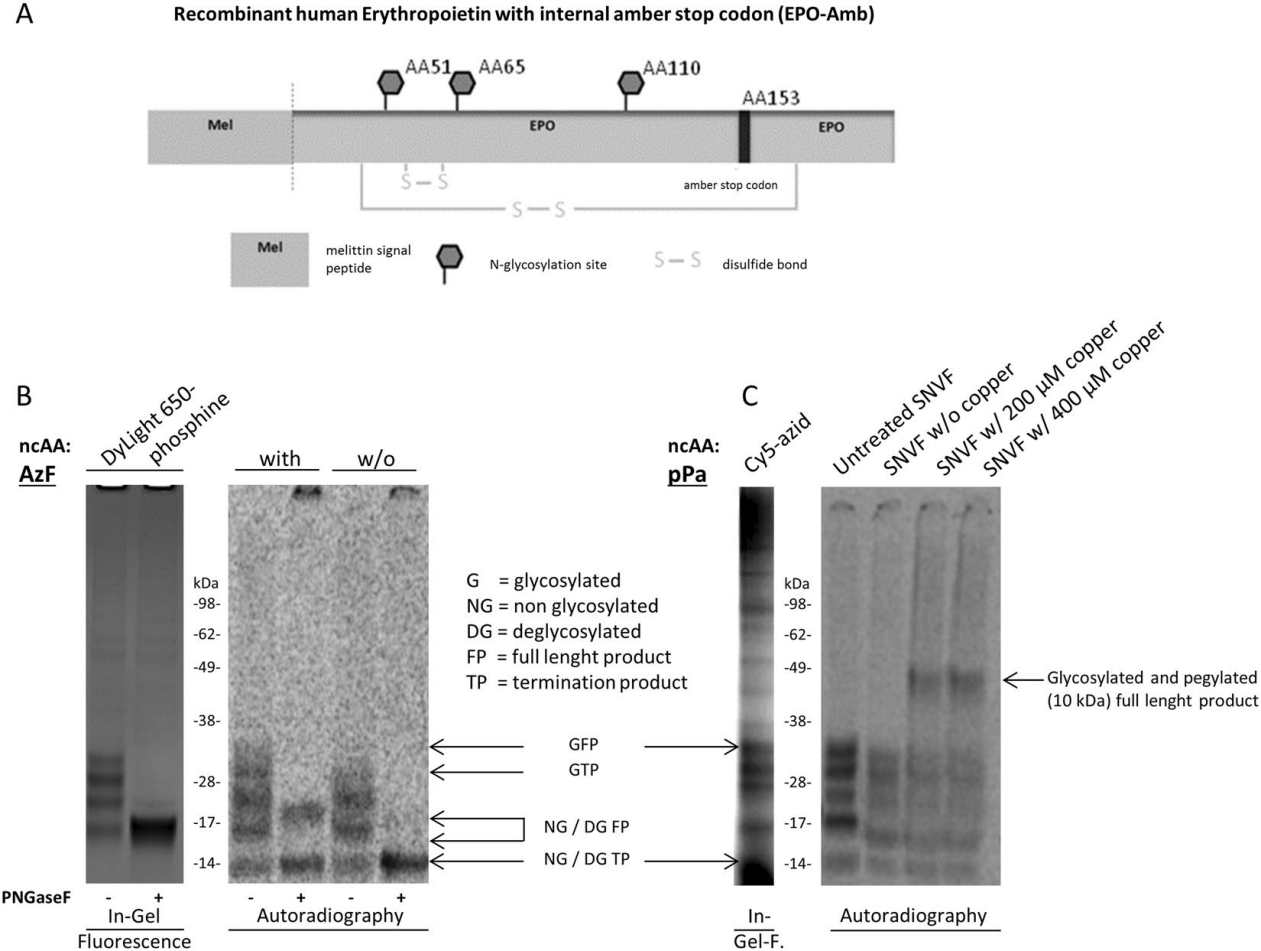
Chemoselective labeling of erythropoietin *amber*-mutants (EPO-Amb). (**A**) Schematic image of EPO-Amb with melittin signal sequence, N-glycosylation sites, disulfide bonds and internal *amber*-stop codon. (**B**) In-gel fluorescence (left image) of DyLight650-Phosphine labeled EPO-Amb after synthesis in the presence of p-azidophenylalanine (AzF). DyLight650-Phosphine sample was afterwards digested with PNGaseF (lane 2). Autoradiography (right image) in the presence (lane 1 and 2) and absence (lane 3 and 4) of AzF and after PNGaseF digestion (lane 2 and 4). In the autoradiography (right image) glycosylated full length product (33 kDa) was detected in the AzF supplemented reaction (lane 1). PNGaseF digestion revealed an additional termination product of 14 kDa (lane 2). Synthesis without AzF led to glycosylated termination product (28 kDa, lane 3). PNGaseF digestion (lane 4) indicates no formation of full length product. (**C**) In-gel fluorescence (left image) of Cy5-sulfo-azid (lane 1) labeled EPO-Amb after synthesis in the presence of p-propargyloxyphenylalanine (pPa). Qualitative analysis of EPO-Amb in the presence of pPa, using autoradiography (right image). The labeling reaction was performed with soluble EPO released from the microsomal lumen (SNVF). After completing the labeling reaction with 10 mM of PEG-azide (molecular weight 10 kDa), the bands of EPO-Amb shift to a higher molecular weight of approx. 43 kDa (lane 3 and 4), indicating PEGylation. In the absence of any copper no shift to a higher molecular weight was detected.

The cell-free environment provides an outstanding advantage in particular for the synthesis of site-specific engineered proteins. Aminoacyl tRNA synthetase/tRNA pairs that act orthogonal to native synthetase/tRNA pairs enable the site-specific incorporation of a given non-canonical amino acid (ncAA) using an internal amber-stop codon within the template sequence. This methodology opens access to broad site-directed protein modifications by applying chemoselective reactions of biomolecules with the incorporated ncAA. In this study we present the synthesis of an EPO analogue with an internal *amber*-stop codon inserted at the original O-glycosylation site (EPO-Amb) which is used for directed, orthogonal incorporation of the ncAAs p-propargyloxyphenylalanine (pPa) and p-azido-L-phenylalanine (AzF) and chemoselective labeling with fluorescent dyes and a PEG-azide, respectively (Fig. 4). The incorporation of Ppa and AzF was proven by fluorescence scanning of chemoselective labeling with Sulfo-Cy5-azide and DyLight650-phopshine (Fig. 4B, left panel). The detection of DyLight650-phosphine signals, both before and after the deglycosylation assay, indicates a successful labeling of the full length product released from the ER-derived microsomes present in the lysate. As a control experiment, synthesis of EPO-Amb in the absence of AzF was performed, where autoradiography showed three bands. In comparison to the full length glycosylated EPO all three bands display a significant lower MW and therefore correspond to the expected different glycosylated forms of the termination product. Moreover the site-specific incorporation of pPa into EPO-Amb (EPO-pPa) was verified by MS/MS (Supplementary S3). Site-specific incorporation of pPa and its chemoselective labeling with PEG is illustrated in Fig. 4C. Labeling of EPO-pPa with PEG10k (MW 10 kDa) via copper dependent click chemistry resulted in a shift of the protein band to a higher molecular weight (lane 3 and 4), indicating a successful labeling reaction. Insertion of AzF and pPa into the EPO-Amb sequence provides a platform for adding desired structures to the protein’s O-glycosylation site via chemoselective labeling.

**Figure 5:**
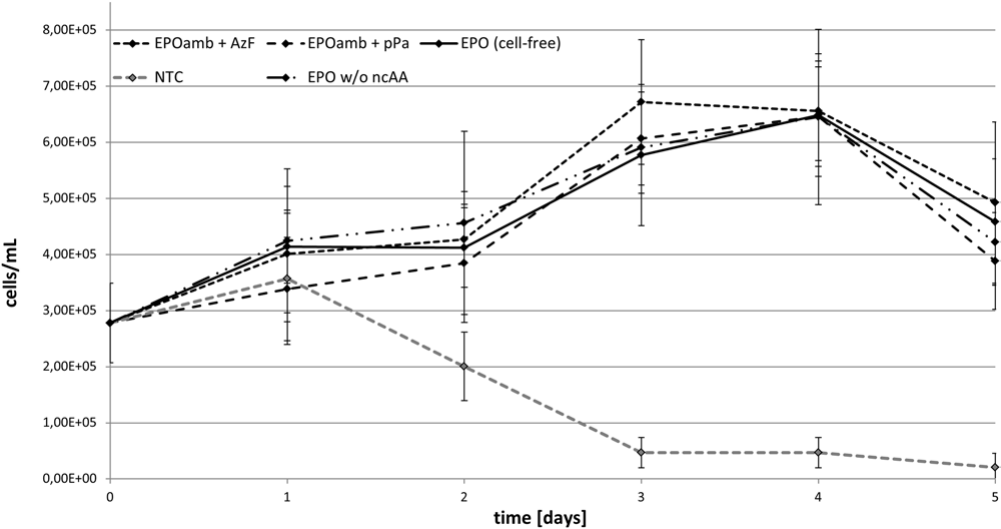
Cell-based activity assay of erythropoietin (EPO) analogues: Growth curves of UT-7 (DSMZ-No. ACC-137) cell-line supplemented with cell-free synthesized EPO (continuous line), cell-free no-template-control (NTC, grey-dashed line), cell-free synthesized EPOamb with incorporated AzF (larger dash-dot line), cell-free synthesized EPOamb with incorporated pPa (smaller dash-dot line) and cell-free synthesized EPOamb in the absence of any ncAA (mixed line). The concentration of cell-free produced EPO was adjusted according to the total protein concentration determined by TCA-precipitation. The NTC led to a continuous decrease of living cells.

Analysis of *in vitro* activity of cell-free synthesized and modified EPO was performed as described above. Cell-growth was monitored for 5 days and the obtained growth curves are presented in Fig. 5. The negative control and the data obtained from unmodified cell-free synthesized EPO correlate to the results previously mentioned (Fig. 3). EPO promoted cell proliferation and the negative control resulted again in a continuous dieback of the cell culture. EPO samples modified either with incorporated AzF or pPa also resulted in a cell proliferation. Interestingly no difference could be detected in cell proliferation when comparing the EPO sample with incorporated non-canonical amino acid to the sample without non-canonical amino acid.

## Discussion

The presented work describes the cell-free protein synthesis of glycosylated EPO, its structural and functional characterization and activity studies. Although glycosylated EPO has been already synthesized in cell free systems^17,18,23^, the process of N-glycosylation in *Sf*21 lysates and the resulting glycan structures attached to the protein backbone have not been investigated yet. In this work, we extend the understanding of the *Sf*21 lysate’s enzymatic machineries, showing that the cell-free system retains the capability to synthesize fresh sugar precursors. The identified glycan pattern HexNac_2_(Hex)_5–8_ are in good agreement with general insect glycosylation, corresponding to the high mannose type HexNac_2_(Man)_5–8_. Previous studies demonstrated the activity of the ER-resident α-glycosidase I/II and mannosidase I in insect cells, which are important for the initial trimming steps from the precursor glycan Glc_3_Man_9_GlcNAc_2_ to Man_8_GlcNAc^24^. Moreover, activity of the *cis*-Golgi-resident enzyme α1-2 mannosidase was shown also in insect cells^25^. This enzyme is important for further trimming from Man_8_GlcNAc_2_ to Man_5_GlcNAc_2_, a key intermediate for further remodeling and the formation of hybrid or complex glycan structures^26^. For further processing of Man_5_GlcNAc_2_ to hybrid or complex glycan structures, N-acetylglucosaminyltransferase I is essential^26^. This enzyme was shown to occur in insect cells, however only low activity could be determined^27^. Therefore the detected HexNac_2_(Man)_5–8_ and the absence of more complex N-glycan structures as they usually appear in human EPO could be explainable by glycan trimming as well as the low activity of N-acetylglucosaminyltransferase I. The fact that a varying number of terminal hexoses were detected (5–8) could result from partly unfinished trimming activities at the time point of protein isolation. Detailes studies of *Sf*21 microsomes, revealed the presence of polypeptide N-acetyl-galactosaminyltransferase. This enzyme is localized in the Golgi and representing an essential component for the initiating step of O-glycosylation^28^. Usually a defined organization of the enzymatic machinery in the Golgi membranes is mandatory, which might not be fully preserved in the microsomes of the *Sf*21 lysates^29^. This might not be critical for the enzymes involved in early processing of N-glycosylation, however it is supposed to be essential for the polypeptide N-acetyl-galactosaminyltransferase and other glycosyltransferases involved in O-glycosylation. Additional enzymes that are required for O-glycosylation were not identified. EPO synthesized in our system was found to be present in several glycoforms, displaying a certain heterogeneity regarding the glycan length. In comparison to mammalian glycosylation, the glycosylation present in our system is clearly less heterogeneous due to a reduced complexity. A comparison between a commercially available rhEPO, expressed in CHO cells and a *Pichia pastoris* derived rhEPO revealed clearly the heterogeneity of glycosylation in a mammalian derived EPO^30^. The MALDI-TOF spectrum depicted a broad peak centered at ~29 kDa with a glycan proportion of 38%. N-glycans were identified as a mixture of sialylated bi-,tri- and tetra-antennary structures and Galactose-N-acetylglucosylamine (LacNAc) extensions in varying positions. The digestion of the complex glycans with neuraminidase and galactosidase to remove sialic acids and galactose resulted in minor complex glycans that are comparable to ER-derived glycosylations (HexNac_2_(Man)_5–8_) as detected in our cell-free system. These minor complex gylcans displayed an increased heterogeneity in comparison to the glycans identified in our study. Nevertheless it has to be mentioned that even after galactosidase digestion occasional galactose residues were identified in the glycan pattern of EPO produced in a cell-based system^30^. Up to date there are only three FDA proved therapeutics available on the market that are produced in insect cells. All of these are vaccines, based on a non-glycosylated protein^31^. The altered glycosylation pattern of proteins produced in insect cells consisting mainly of high mannose and paucimannose types may be one of the reasons for their low number in FDA approved drug products. The limited glycosylation capacity of insect cells might result in proteins displaying a reduced activity, shortened life-time and immunogenicity. Furthermore a α1.3-linked fucose might have allergenic effects^32^. Moreover sialic acids are usually absent in insect cells. Nevertheless similar problems can be found in several nonhuman mammalian cell lines which possess a different kind of sialic acids and different linkage between individual sugar moieties. To partly circumvent the mentioned limitations the analyzed glycans in this study (HexNac_2_(Man)_5–8_) might be substituted with sialyloligosaccharides. Therefore the high mannose type is an ideal substrate for Endo-H digestion, leaving a terminal GlcNac at the asparagine residue at each N-glycosylation site. An engineered glycosynthase based on Endo-M (EndoM-N175A) is known to be able to transfer N-glycans, including sialylated biantennary complex types based on an oxazoline derivate to a single GlcNAc residue^33^. For a further improvement of the glycan transfer to the GlcNAc residues a second acceptor substrate composed of a core focusylated EPO glycoform was investigated (Fucα1,6GlcNAc-EPO). The transfer of the sialylated biantennary complex types based on an oxazoline derivate carried out by an engineered EndoF (EndoF3-D165A) resulted in two to three occupied N-glycosylation sites with a high conversion efficiency^33^.

Nevertheless it is of outstanding interest to further improve the cell-free *Sf*21 system in order to produce high glyco protein yields. In this study a protein yield of 5 µg/ml EPO (vesicular fraction) was obtained which is in line with previously reported yields^34^. Reported protein yields of EPO synthesized in an insect based drosophila S2 cell line are in the range of 18 µg/ml^35^. The company Cell Culture Technologies supplies an engineered CHO cell line secreting EPO with a typical EPO concentration up to 50 µg/ml. Production of EPO in *E. coli* resulted in yields of 129 µg/ml before^36^ and 7.2 µg/ml after purification^37^.

High protein yields might be realized by optimization of the lysates, in terms of cultivation requirements of the initial cell culture^38^, cell disruption technologies and protein synthesis conditions^34^. Another option might be the introduction of an IRES site into the template DNA in combination with its application in a dialysis reaction mode. It has previously been shown that this combination can increase the protein yield in the case of a transmembrane protein dramatically^39^. In addition to optimizing protein yields, for a production aspect the scalability should be considered. The presented work was mainly performed in a small scale of 20–50 µl. An upscaling to a volume of 500 µl showed no significant variation in protein yield (Fig. S4). The linearly scalability of a cell-free system based on *E. coli* lysates was shown in 2011 by the production of a granulocyte-macrophage colony-stimulating factor in a volume up to 100 L^40^. In order to optimize the glycosylation potential of the eukaryotic cell-free system, an increase of the percentage of vesicles in the lysate as well as a supplementation with additional sugar building blocks might be beneficial. In this context variations in the glycan pattern of cell-free produced EPO can be detected (compare Fig. 1A with B). In Fig. 1A bands of different molecular weight can be seen, corresponding to a partly (one and two occupied glycosylation sites), a fully (three occupied glycosylation sites) and a non-glycosylated EPO. Although the predominant band corresponds to the fully glycosylated EPO a large proportion is only partly glycosylated. In contrast a different glycosylation pattern can be seen in the radioactive mannose labeling (Fig. 1B), where no partly glycosylated EPO was detected. This difference might result from a competition between endogenously present mannose in the system and the radioactively labeled mannose. For the detection of the ^14^C-mannose a certain concentration of radioactively labeled mannose has to be present in the glycosylation pattern. This concentration might only be reached with three occupied glycosylation sites.

In addition these approaches may give rise to a repeated targeting of the microsomes with e.g. specific glycosyltransferases to achieve modulated glycosylation patterns on a desired protein.

The open nature of the cell-free eukaryotic translation system offers the possibility for straightforward site-specific incorporation of ncAAs via *amber* suppression and subsequent chemoselective labeling^41^. This methodology is of particular interest in the field of protein engineering. By expanding the natural repertoire of building blocks, a higher diversity of AA (non-canonical as well as canonical) is available to directly alter the protein´s characteristics. Nevertheless the cotranslational incorporation of ncAAs is limited by the steric effects of the ribosome. Chemoselective labeling however offers the opportunity to modify site-specifically incorporated ncAAs with nearly any biomolecule containing the appropriate functional group for the reaction. This procedure is of particular interest for pharmaceutically relevant proteins, such as hEPO, who’s *in vivo* bioactivity and half-life strongly depends on its glycosylation pattern^42^. Different posttranslationally modified hEPO analogues such as darbopoietin alfa and CERA were already developed, resulting in significant improvements in pharmacological efficacy^43,44^. We describe the synthesis of EPO-Amb, an analogue with an internal *amber*-stop codon. Site-specific incorporation of two different ncAAs, embodying either an azide or an alkyne group, was achieved. In addition, subsequent attachment of different labels to these ncAAs in the protein backbone using both, copper dependent click chemistry as well as Staudinger ligation was demonstrated. Interestingly modified EPO showed no difference in inducing proliferation in the absence or presence of any non-canonical amino acids. This result indicates that a possible termination product might be able to induce cell-proliferation as well. The binding mechanism of EPO to its receptor can be divided into two parts^45^. EPO binds with high affinity (1 nM) to the first receptor monomer (site 1), thus inducing a dimerization and a subsequent binding with a lower affinity (1 µM) to the second receptor molecule (site 2). The binding to site 2 is promoted by EPO residues in helices A and C and the major contributors to this binding are the residues Lys124, Ser127, Arg130, Ser131, Thr134 and Leu135^46^. EPO residues Arg130, Ser131, Leu132, Leu135 and Arg137 were identified to be responsible for cell proliferation^45,47,48^. These residues are maintained in the termination product. Moreover a publication from 2010 utilized a peptide containing the EPO residues 119–138 to develop a non-haematopoietic agonist of the EPO-receptor^49^. In this study the specific binding of the shortened peptide to the EPO-receptor was shown. Moreover the proliferation of a growth factor dependent cell line was promoted by adding 5 µM of the peptide. Therefore it might be possible that the termination product (protein fragment including the first 153 amino acids) is sufficient to induce cell proliferation as well. In this context the termination product has a missing disulfide bond (Cys_34_-Cys_188_). The formation of disulfide bonds in cell-free systems has been reported previously^19,50^. Early studies of EPO revealed that the disulfide bond between Cys_29_ and Cys_33_ has no significant influence on the functionality. In contrast the intramolecular disulfide bond between Cys_34_-Cys_188_ seems to be crucial for the preservation of the molecular structure^51^. Nevertheless studies with an initial reduction of Cys_34_-Cys_188_ disulfide bond showed a partial dimerization of EPO monomers exhibiting biological activity^52,53^. It might be possible that the cell-free produced termination product with an unoccupied cysteine residue, showed a similar behavior in our experimental setup. The positive influence of polyethylene glycol chains on the solubility, stability and activity of non-glycosylated EPO, produced in *E. coli*, was shown recently^54^. In this study single N-glycosylation sites were substituted with short PEG chains (750 Da/2000 Da) by using *amber* suppression. Each of the three substituted N-glycosylation sites with PEG resulted in an active protein. The attachment of larger PEG chains (4000 Da) resulted in EPO variants with a comparable or even higher bioactivity as the mammalian derived EPO used as a positive control.

This gives rise for further modifications e.g. based on chemically synthesized glycan-azides^29^ in order to study the functional effect of the *de novo* designed and homogenous glycosylation patterns on the protein’s activity and stability. The obtained results underline the potential of the insect-based cell-free system for glycoengineering of recombinant EPO. A forward-looking development might be the adaption of this glycoengineering methodology to a mammalian cell-free system. This might be beneficial for future industrial EPO production.

## Electronic supplementary material

Supplementary Information
