# Supplementary material for: Cell-free protein synthesis as a novel tool for directed glycoengineering of active erythropoietin
Source: Sci Rep. 2018 Jun 4;8:8514. doi: 10.1038/s41598-018-26936-x (PMC5986796; doi:10.1038/s41598-018-26936-x)
Supplement: Supplementary file 1 — Supplementary Information [file 41598_2018_26936_MOESM1_ESM.pdf]

Cell-free protein synthesis as a novel tool for directed glycoengineering of active erythropoietin

Anne Zemella[1], Lena Thoring[1], Christian Hoffmeister[1], Mária Šamalíková[1], Patricia Ehren[2], Doreen A. Wüstenhagen[1] and Stefan Kubick[1*]

[1] Fraunhofer Institute for Cell Therapy and Immunology (IZI), Branch Bioanalytics and Bioprocesses (IZI-BB), Potsdam Golm, Germany

[2] University of Potsdam, Am Mühlenberg 11, 14476 Potsdam-Golm, Germany

[*] Corresponding author: stefan.kubick@izi-bb.fraunhofer.de

Keywords: Cell-free protein synthesis, erythropoietin, glycosylation, microsomes, chemoselective labeling, mass spectrometry

Supplementary

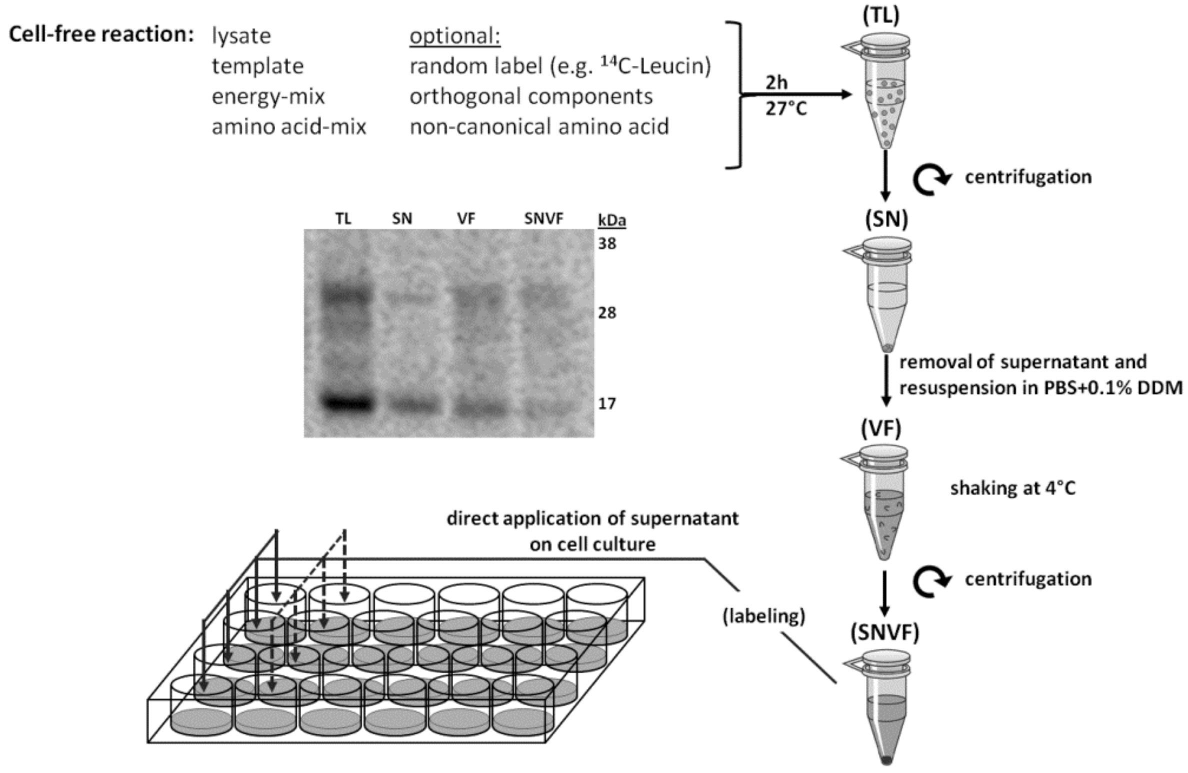

S1: Work flow of protein release from microsomes after cell-free synthesis. The translation mixture (TL) was centrifugated immediately after the synthesis. The supernatant (SN) was removed while the pelletized microsomes (vesicular fraction, VF) were resuspended in PBS+0.1 % DDM in order to perforate the vesicle membranes. After vigorous shaking, another centrifugation step separated membrane debris and associated proteins in the pellet from the released soluble proteins in the supernatant (supernatant of VF, SNVF). This fraction was directly used for labeling reactions and thus was applied in the cell based activity assay. Autoradiography of the different fractions visualized that majority of the glycosylated erythropoietin of the VF could be released into the SNVF with this method.

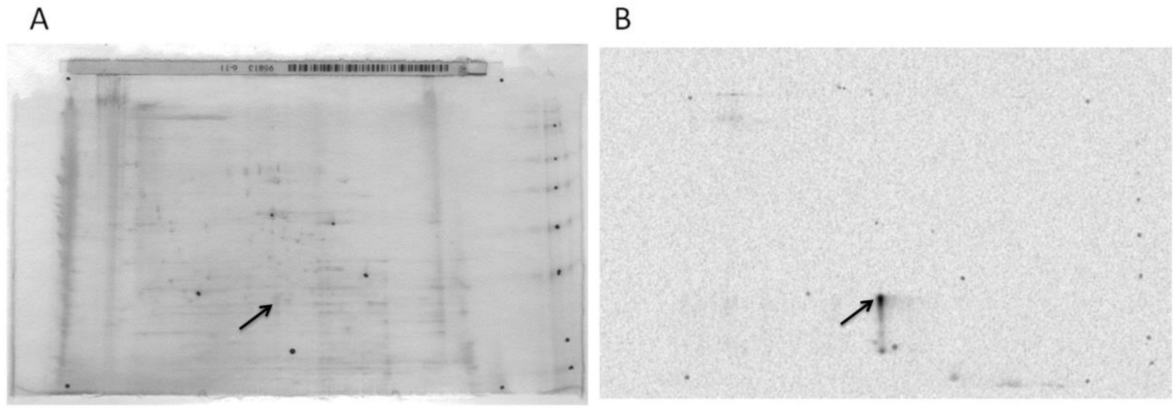

S2: 2D-Gelelectrophoresis of cell-free synthesized erythropoietin (EPO) in the vesicular fraction of the translation mixture. A: Coomassie stained 2D-gel of vesicular fraction of ^{14}C-labeled, cell-free synthesized EPO. The marker bands as well as some protein spots were labeled with radioactive ink in order to localize EPO in the gel. B: Autoradiography image of scanned 2D-gel.

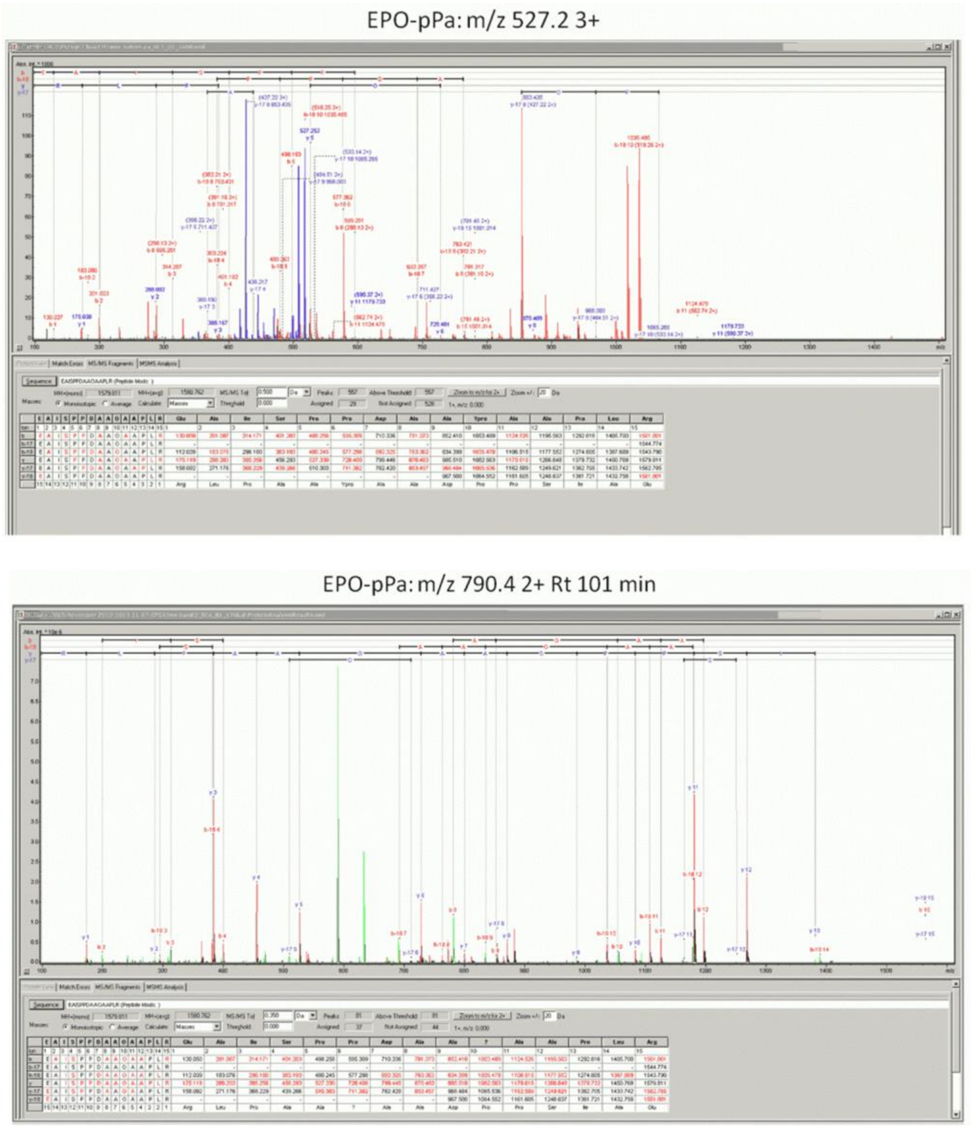

S3: Collision-induced dissociation tandem masspectrometry spectra of cell-free synthesized EPO-Amb with incorporated p-propargyloxyphenylalanine (pPa). pPa is referred to as O in the spectra. Both spectra show the incorporated non-canonical amino acid pPa at the expected site regarding the *amber*-mutation of its DNA. A: m/z 527.2 (3+) with annotation of *b* and *y* series B: m/z 790.4 (2+) with annotation of *b* and *y* series.

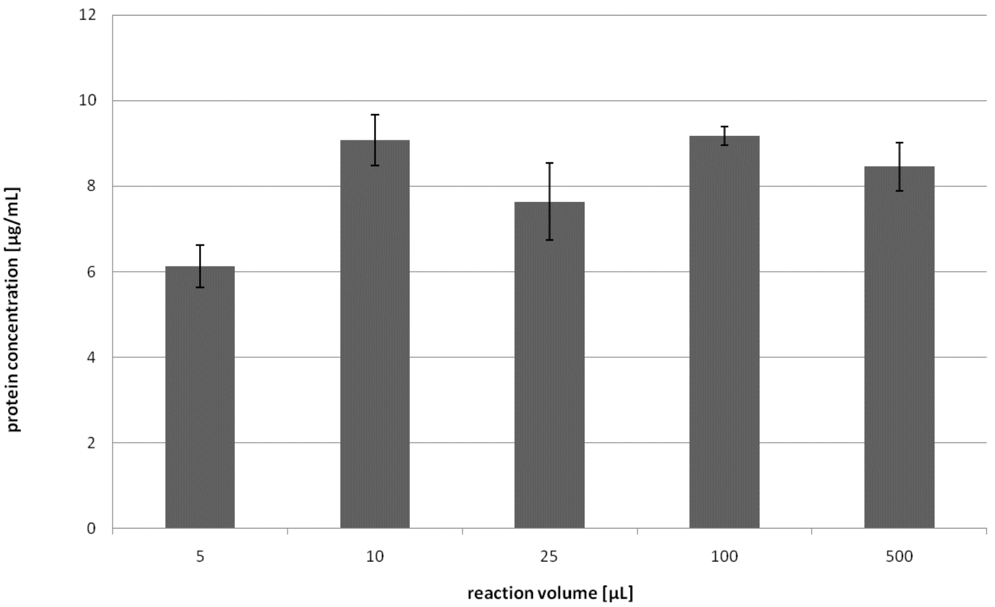

S4: Upscaling of synthesis of EPO in an insect-based cell-free system. The protein yield of cell-free synthesized EPO in different reaction volumes was analyzed. Reactions were carried out in a standard Eppendorf tube and incubated for 2 h at 27 °C with gentle shaking. An upscaling to a volume of 500 µl showed no significant variation in protein yield
